# Polyethylene glycol (5,000) succinate conjugate of lopinavir and its associated toxicity using *Danio rerio* as a model organism

**DOI:** 10.1038/s41598-020-68666-z

**Published:** 2020-07-16

**Authors:** Oluwole Samuel Aremu, Lebogang Katata-Seru, Zimbili Mkhize, Tarryn Lee Botha, Victor Wepener

**Affiliations:** 1 Chemistry Department, Faculty of Natural and Agricultural Sciences, North-West University, Mmabatho, 2735 South Africa; 2 Water Research Group, Unit for Environmental Sciences and Management, North-West University, Private Bag X6001, Potchefstroom, 2520 South Africa

**Keywords:** Chemistry, Medicinal chemistry, Drug discovery and development

## Abstract

Lopinavir (LPV), a well-known drug administered in human immunodeficiency virus (HIV) infection, has shown limitation for pediatric treatment owing to poor aqueous solubility that gives rise to limited oral bioavailability and short plasma half-life (5–6 h). Polymers such as polyethylene glycol (PEG) have been used as drug carriers to improve their solubility. This study reports the preparation of polyethylene glycol (5,000) succinate (PEG–Suc–LPV) conjugate of LPV by the esterification method. The disappearance of the 3,395 cm^−1^ (O–H stretch of COOH) band for Polyethylene glycol (5,000) succinate (PEG–Suc )confirms the formation ester linkage with the OH group of LPV which is also confirmed by ^1^H NMR analysis. The XRD for the conjugate showed a broad, amorphous peak while pure PEG, Suc, LPV are crystalline. DSC analysis showed that the conjugate exhibited new broad and diffuse peaks, confirming that they did exist in an amorphous state as multiple complexes. The conjugate showed improved solubility and activity with reduced toxicity compared to pure LPV. The solubility of LPV increased significantly from 80 to 318 ppm. Furthermore, an aquatic toxicity test using *Danio rerio* showed that the conjugate had a lower LC_50_ (60.8 ppm) when compared to the pure LPV drug LC_50_ (6.42 ppm). These results suggest PEG–Suc conjugate of LPV as an efficient carrier for enhanced hydrophilicity and anti-HIV property of LPV.

## Introduction

The advancement of antiretroviral (ARV) therapy has managed to increase the lifespan of people living with human immunodeficiency virus (HIV). Recently, it was reported that 23.3 million people since 2018 were receiving antiretrovirals (ARVs) compared to 21.7 million in 2017^1^. However, some of the critical challenges facing HIV treatments includes low aqueous solubility, extensive first-pass metabolism, low bioavailability, and lack of patient adherence. An illustrative example is the orally administered lopinavir (LPV) drug that has shown low bioavailability because of poor aqueous solubility, limited intestinal uptake caused by efflux, and cytochrome P450^2,3^. Hence the drug is always co-administered with ritonavir^3^.

Novel nanoformulation approaches to enhance the oral bioavailability of LPV have been investigated by various researchers^4–8^. Patel made an LPV propliposome presenting a vesicle of 659.7 nm Patel, et al. 4 fabricated LPV propliposome exhibiting a vesicle of 659.7 nm. Their obtained results showed a higher oral bioavailability in rats as compared to pure LPV suspension. The development of pegylated drug conjugates has gained an interest in ARVs^9^. Li synthesized pegylated zidovudine conjugates, and their pharmacokinetic studies showed an increased bioavailability and a 2–threefold prolonged half-life compared to free AZT.

Hence, this study report for the first time the synthesis and characterization of PEG–Suc–LPV. Besides, its solubility and their toxicity studies with *Danio rerio*, Zebrafish. Zebrafish have been studied extensively and are seen as an internationally accepted model using OECD (eco) toxicology studies based on their close homology with the human genome^10^. Since a significant primary focus of this study based on increasing the solubility of LPV, the use of zebrafish played a complementary role as an appropriate effect-based monitoring tool under the water framework directive^11^.

## Methods

PEG with two hydroxyl terminals and MW of 5 kDa-unit, succinic anhydride (SA), *N,N*’-diisopropyl carbodiimide (DIC), 4-dimethyl aminopyridine (DMAP), triethylamine (TEA), dichloromethane, dimethyl sulfoxide (DMSO), acetonitrile and dimethylformamide (DMF) were all purchased from Sigma Aldrich (South Africa).

### Synthesis of PEG–Suc 5 kDa

**Scheme 1:**
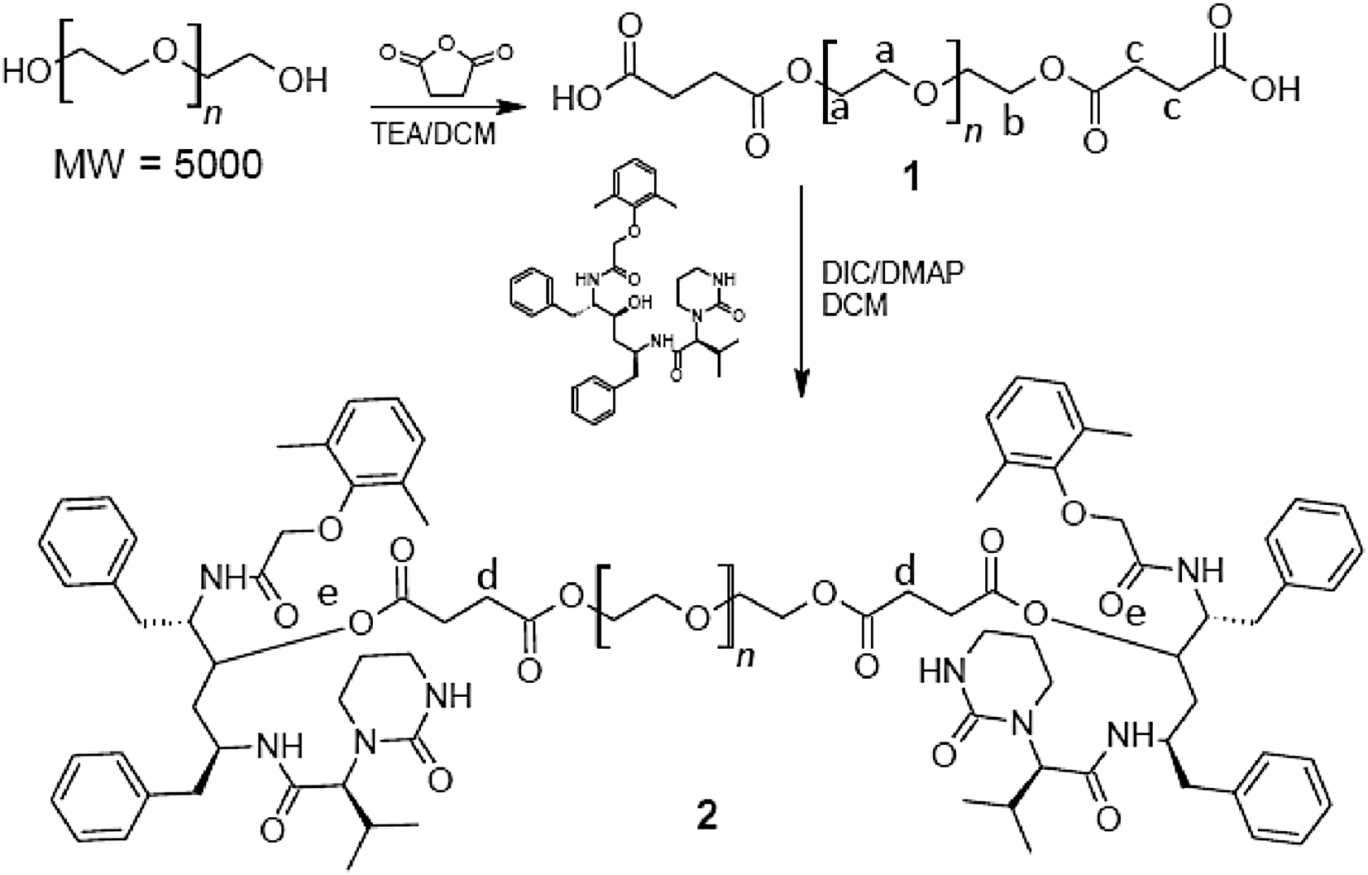
Synthetic scheme of the succinate derivatives pegylated lopinavir.

The synthesis of Pegylated Succinate (PEG–Suc) was accomplished following a modified method based on that of Luo et al.^12^. PEG 5 kDa (100 mg, 0.200 mmol, 1.0 equiv.) was dissolved in 2 mL toluene and evaporated three times to remove the water, with the dried PEG dissolved in dichloromethane under nitrogen. The solution cooled to 0 °C, SA was added (10 mg, 0.0899 mmol, 6.0 equiv.), and TEA (0.14 µL, 0.098 mmol, 6.0 equiv.) was dropped with a syringe. The reaction was then kept on ice for 60 min and was stirred at 25 °C for 24 h. The PEG–Suc was purified by precipitation in cold diethyl ether three times, the precipitates were dried under vacuum at room temperature overnight, and the yield was 95% at PEG MW (Scheme 1(**1**)).

### Synthesis of PEG–Suc–LPV conjugates 5 kDa

The synthesis of Pegylated–Succinate–Lopinavir conjugate, (PEG–Suc–LPV) was prepared using a modified method based on that of Luo et al.^12^. PEG–Suc, 5 kDa (100 mg, 0.200 mmol, 1.0 equiv.), LPV (37 mg, 0.0387 mmol, 2.5 equiv.), and DMAP (5 mg, 0.0.397 mmol, 2.5 equiv.) were dissolved in 1.5 mL DCM under nitrogen, with the solution placed on ice and DIC (2.0 μL, 0.0390 mmol, 2.5 equiv.) being added dropwise into the flask, the reaction was stirred for 24 h at room temperature, and the conjugate was purified by precipitation in cold diethyl ether three times and dried under vacuum. (Scheme1 (**2**)).

### Attenuated total reflection Fourier transform infrared (ATR-FTIR) spectroscopy

Cary 600 Series ATR-FTIR spectrometer over the range 4,000–400 cm^−1^ were used to record the spectra of the conjugates' compatibility to study the interactions between PEG, SA, LPV, PEG–Suc, and PEG–Suc–LPV. The samples were placed directly in the FTIR sample holder in direct contact with the total reflection accessory crystal and infrared spectra in transmittance mode^13^.

### Thermal analysis

The sample thermal properties were determined on an indium-calibrated Mettler Toledo differential scanning calorimeter (DSC, Perkin Elmer, Überlingen, Germany), with an initial heating scan of − 10 to 100 °C, a cooling scan of 100 to − 10 °C and another heating scan of − 10 to 100 °C at a scan rate of 10 °C/min. Crystallinity was assumed to be proportional to the experimental heat of fusion of 139.5 J g^-^1 for the 100% crystalline samples^14^.

### X-ray diffraction (XRD)

The X-ray powder diffraction patterns were obtained using a PANalytical Empyrean diffractometer (PANalytical, Almelo, Netherlands), the measurement conditions being: target, Cu; voltage, 40 kV; current, 30 mA; divergence slit, 2 mm; anti-scatter slit, 0.6 mm; detector slit, 0.2 mm; monochromator; scanning speed, 2°/min (step size, 0.025°; step time, 1.0 s)^15^.

### Solubility studies

Solubility determination of the conjugates and the drug were added in vials containing distilled water, it was shaken for 24 h at room temperature in a bath with a shaker until equilibrium is reached. The resultant suspension was then filtered through Whatman filter paper No. 1 and suitably diluted with distilled water. Finally, the filtrate was analyzed by UV. 100 to 900 mg/ml of the pure drug were prepared, and the absorbances measured at 257 nm using a Perkin Elmer Lambda 365 UV–Visible recording spectrophotometer. A standard curve was plotted (R^2^ 0.9975) using the acquired data from the absorbance of the filtered samples, then solubility was calculated from the values obtained from the standard curve of the pure drug^16^.

### Toxicity studies using *Danio rerio*

All wild type zebrafish used within this study were maintained in a Tecniplast ZebTec system at the National Aquatic Bioassay facility at North-West University, with the juvenile fish used within this study being bred two weeks before testing. The South African National Standards (SANS, 2008: 10,386^17^ for the care and use of animals for scientific purposes) was followed for all husbandry and breeding. The fish acute lethality test^11^ was performed on 14-day old juveniles following OECD protocols, according to ethical procedures as approved by the AnimCare Committee at North-West University (NWU-00269–16-A5).

The standard OECD reconstituted water contained CaCl_2_.2H_2_O, MgSO_4_.7H_2_O, NaHCO_3,_ and KCl (Sigma Aldrich, South Africa) and was aerated for 24 h before use to obtain at least 60% oxygen saturation. Briefly, three 500 mL beaker replicates containing seven fish per beaker were set up per concentration tested. Both LPV and PEG–Suc–LPV conjugate were prepared in ultrapure water at 100 mg/L stock concentrations and sonicated in a bath sonicator filled with 900 mL of water at 25 °C for one hour. Exposure concentrations were made up in a reconstituted OECD medium in a range of 0.1–40 mg/L. A 12 h light and 12 h dark cycle was maintained for the duration of the test, with the temperature in the room being set to 28 °C.

Physico-chemical parameters were recorded every 24 h, following standard protocols. The temperature, pH, electrical conductivity, and total dissolved solids were measured using a handheld Eutech pH 110 RS232C meter, Eutech CON 110 RS232C conductivity, and TDS meter. Mortality per beaker was recorded at every 24 h interval and was regarded when there was no visible gill movement. For a test to be valid, the control had to have less than 10% mortality for its full duration. A dose–response function was obtained, and a lethal concentration (LCx) value was calculated using the regression analysis (linear or non-linear) in Toxrat Professional. The results were presented as an LC10 (where 10% of the organisms were affected), an LC20 (20% affected), and LC50 (50% affected).

## Results and discussion

### Characterization of PEG–Suc–LPV Conjugate

#### Nuclear magnetic resonance

**Figure 1:**
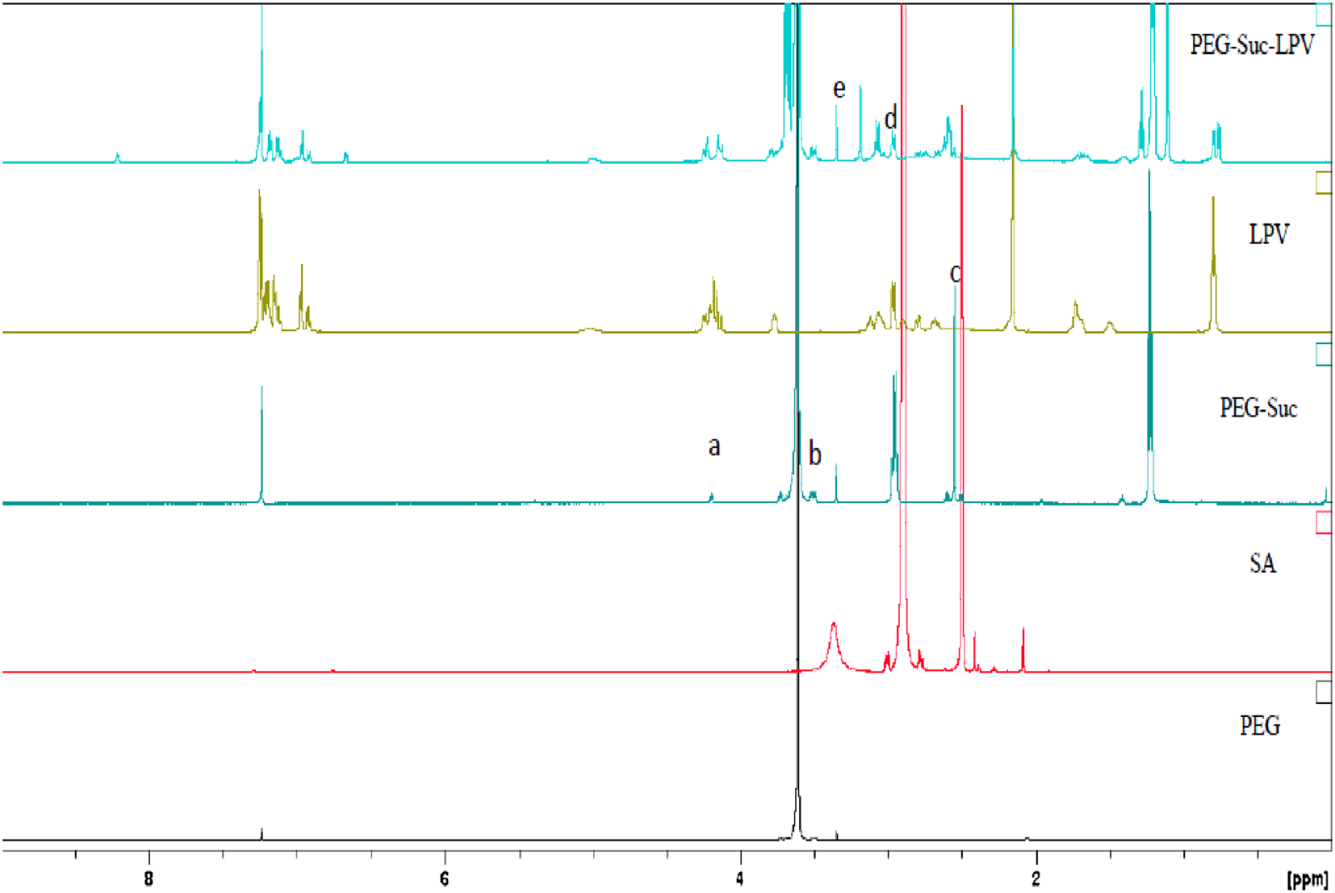
Proton NMR of the PEG–Suc–LPV conjugate and its different starting materials.

Figure 1 showed the proton NMR of the PEG–Suc–LPV conjugate and its different starting materials. The ^1^H NMR 7.24 and 1.56 ppm appear for the chemical shift of the solvent peaks of CDCl_3_, with the proton assignment of the PEG–Suc shown in Fig. 1. Peak “a” (4.19 (t, J = 10.0 Hz, 4H, PEG-O-CH_2_-CH_2_-O-SA), “b” (3.45–3.53 m, PEG backbone), and “c” (2.60 ppm m, 8H, PEG-COO-CH_2_-CH_2_-COOH) indicate conjugation of an ester bond between PEG and SA. In the next step, the PEG–Suc conjugated to LPV via the formation of ester bonds, was also confirmed by ^1^H NMR. Peak “e” shows the ^1^H NMR spectra of PEG–Suc–LPV, which exhibited the characteristic peaks of PEG and LPV. The ^1^H NMR results indicated that the C-3 hydroxyl group of LPV underwent esterification. Compared to the LPV alone (peak “d”), covalent conjugation to the PEG with ester bonds of several different Anti-HIV drugs has been reported to confer improved antiretroviral efficacy and decreased toxicity^18^.

#### ATR-FTIR

**Figure 2:**
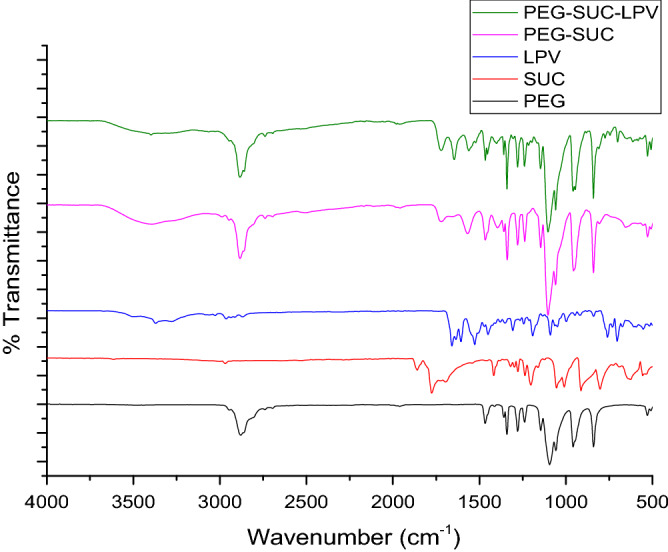
ATR-FTIR of PEG, Suc. PEG–Suc, LPV and PEG–Suc–LPV.

The FTIR spectra of PEG, SA, LPV, PEG–Suc, and PEG–Suc–LPV conjugates shown in Fig. 2, with the characteristic peaks assignments of PEG being 3,411 cm^−1^ (O H stretch), 2,877 (CH), 1,466 cm^−1^ (CH_2_ vibrational stretching for methylene group). The IR spectral bands of SA are 3,616 cm^−1^ (O H stretch), 2,972 cm^−1^ (CH), 1722 cm^−1^ (C=O). The IR spectral band of PEG–Suc: 3,395 cm^−1^ (O H stretch of COOH), 2,884 cm^−1^ (CH), 1718 cm^−1^ (C=O stretching of ester), 1567 cm^−1^ (C O stretch of COOH). The appearance of COOH confirms that the PEG successfully reacted with SA.

The PEG–Suc–LPV conjugates revealed characteristic absorption peaks at 3,397 cm-1 (NH vibrational peak), 2,884–2,739 cm^−1^ (C–H stretch of PEG chain), which were stronger and sharper than the LPV and PEG–Suc. The peaks at 1721–1645 cm^−1^ (C–O stretching of ester), and 1,147–1,103 cm^−1^ (bridge C–O–C stretch) also found in these spectra, but peaks at 3,395 cm^−1^ (O–H stretch of COOH) and 1718 cm^−1^ (C–O stretch of COOH), which were observed in Fig. 2, both peaks disappeared, indicating the COOH of PEG–Suc had conjugated with the OH of LPV. The above FTIR analysis clearly showed that PEG–Suc–LPV successfully synthesized and it is expected that PEG conjugates will act as prodrugs and dissolution of polyethylene glycol moieties leads to release of parent drug l LPV in body fluids without effecting required therapeutic effect. the result above being like that reported by Hifumi et al.^13,19^.

### Differential scanning calorimetry

**Figure 3:**
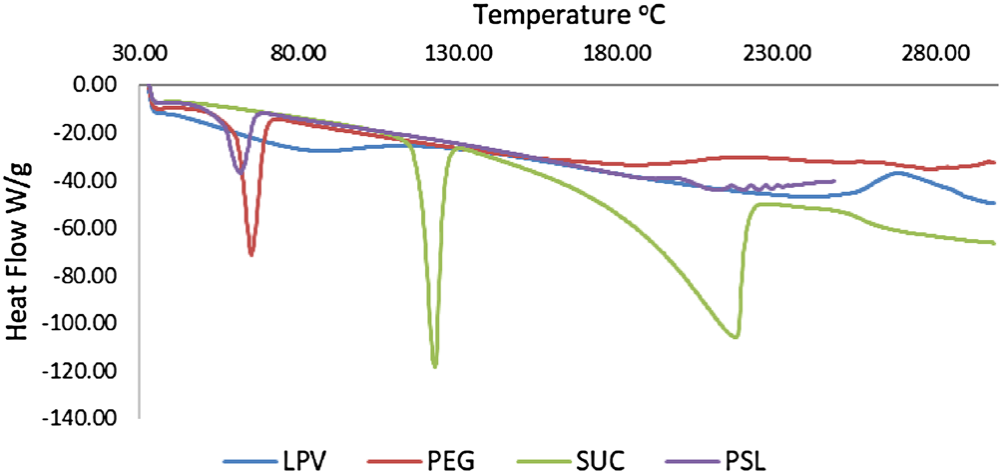
DSC thermograms of PEG, Suc, LPV and PEG–Suc–LPV.

In the DSC, the samples heated at a constant rate of 10 °C and the amount of energy required detected. The pure LPV thermogram showed an endothermic peak at 88.4 °C, which indicates the crystalline nature of LPV. The PEG–Suc–LPV conjugate thermogram showed an endothermic peak at 60.7 °C but not at 88.4 °C. Crystallinity reduction occurs after the decline in thermal temperature and melting point. When the crystallinity decreases, the crystalline nature was converted to amorphous structures, which resulted in solubility improvement (Fig. 3)^20^.

### Thermogravimetric analysis

**Figure 4:**
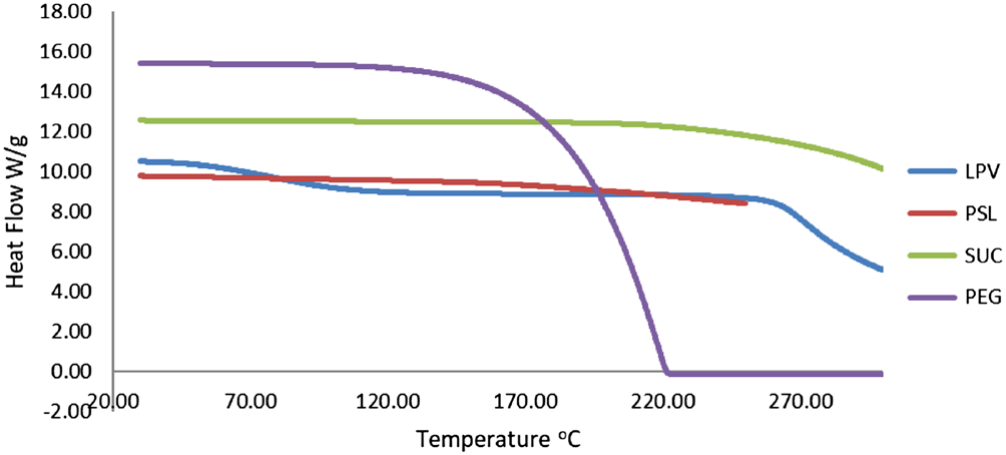
TGA thermograms of PEG, Suc, LPV, and PEG–Suc–LPV.

As shown in Fig. 4, the TGA thermograms of pure LPV showed a two-step weight loss at 80 °C and decomposition at 268 °C compared to the PEG–Suc–LPV, which showed heat stability until 248 °C. This is due to the amorphous phase of the PEG–Suc–LPV compared to the crystallinity phase of the pure LPV, this further confirming the improvement of the solubility of LPV. The PEG showed a complete decomposition between 155–180 °C and a complete degradation at 220 °C, while the Suc showed one step of weight loss at 280 °C (Fig. 4)^21^.

### X-ray diffraction

**Figure 5:**
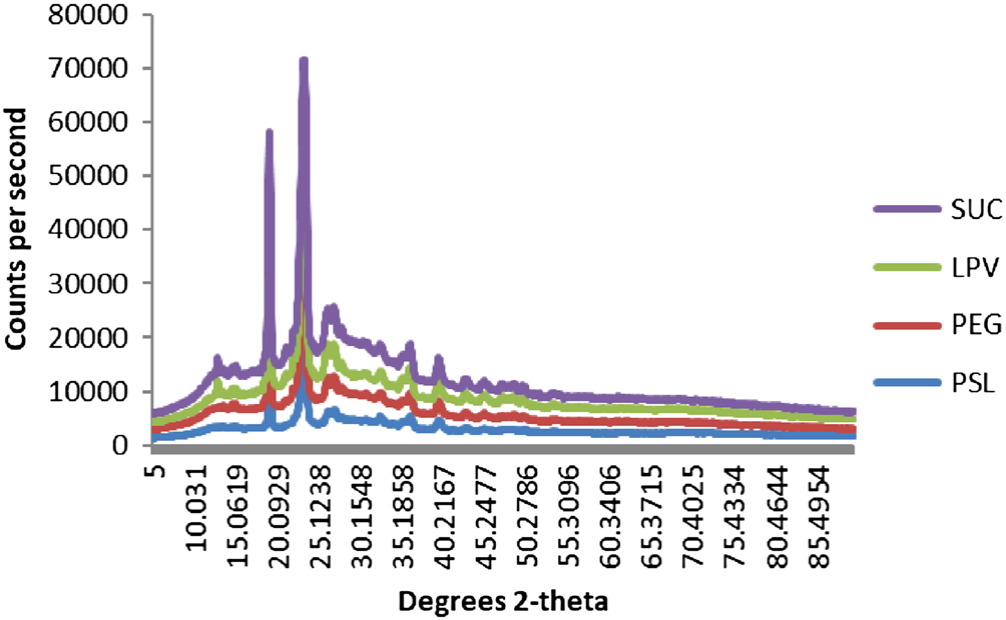
XRD image of PEG, Suc. LPV and PEG–Suc–LPV.

To ascertain the physical state and crystallinity of LPV in the polymeric conjugate, the XRD pattern of pure PEG, Suc, LPV, and PEG–Suc–LPV conjugate are presented in Fig. 5. As can be seen, pure PEG, Suc, LPV are highly crystalline and showed several diffraction peaks at 2ϴ = 15.20, 21.3, 28.13, 29.68, 30 52, 39.12, 40.31 deg, while the diffraction peaks of conjugate were not recorded at the same position, which showed less diffraction peaks with low reduction in crystalline peaks. Formation of new peaks in the conjugate might be attributed to a polymorph structure transformation due to the conjugation of PEG, Suc, and LPV. The conjugate showed a broad amorphous peak. This corresponds to the improved solubility of the conjugate^22^.

### Solubility studies

The results of the solubility studies are shown in Table 1. It was examined in four different media: aqueous, 0.1 N HCl (1.2), PBS (7.4), and MeOH. As indicated, pure LPV shows lower solubility of 80 ppm in water compared to solubility in other media with 847, 626, 555 (ppm). In contrast, PEG–Suc–LPV showed an improved solubility of sixfold in water, threefold in 0.1 N of HCl, fourfold PBS, and tenfold in MEOH compared to free LPV. This enhanced solubility can be attributed to the effect of the formation of amorphous conjugate^23^.

**Table 1:** Saturation Solubility studies of LPV and conjugate (ppm).

Batch	Water	0.1NHCl ((1.2)	PBS (PH 7.4)	MEOH
LPV	80	847	626	555
Conjugate	318	2,231	2,531	523

### Toxicity studies using *Danio rerio*

The reconstituted water was maintained at a constant temperature across all test beakers (26.9 ± 0.16 °C), and the pH remained in a close range (7.93 ± 0.05) for the duration of the test. Although, the control and LPV showed a similar range of total dissolved solids (with 841.3 ± 32.8, ppm and 820.7 ± 13.2 ppm respectively), a difference was seen with, the conjugate having a lower value of 536.5 ± 11.7 ppm. The conductivity followed the same pattern, where the control was 1,202.2 ± 40.8 µS, LPV 1,184.2 ± 22.9 µS and the conjugate 536.5 ± 11.7 µS. This phenomenon can be further related to the solubility seen in different media (Table 1) which shows that as the conjugate reacts with the salts within the reconstituted water, the solubility of the conjugate increases and the salts become unavailable, resulting in a lower conductivity reading within the water medium.

**Figure 6:**
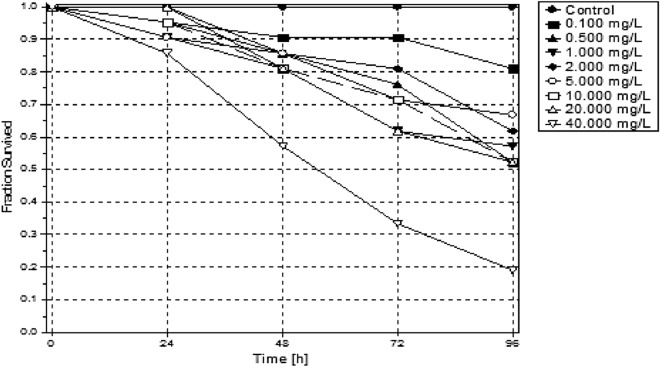
The survival of the introduced 14-day old *Danio rerio* as observed over a 96-h exposure to varying concentrations of LPV.

**Figure 7:**
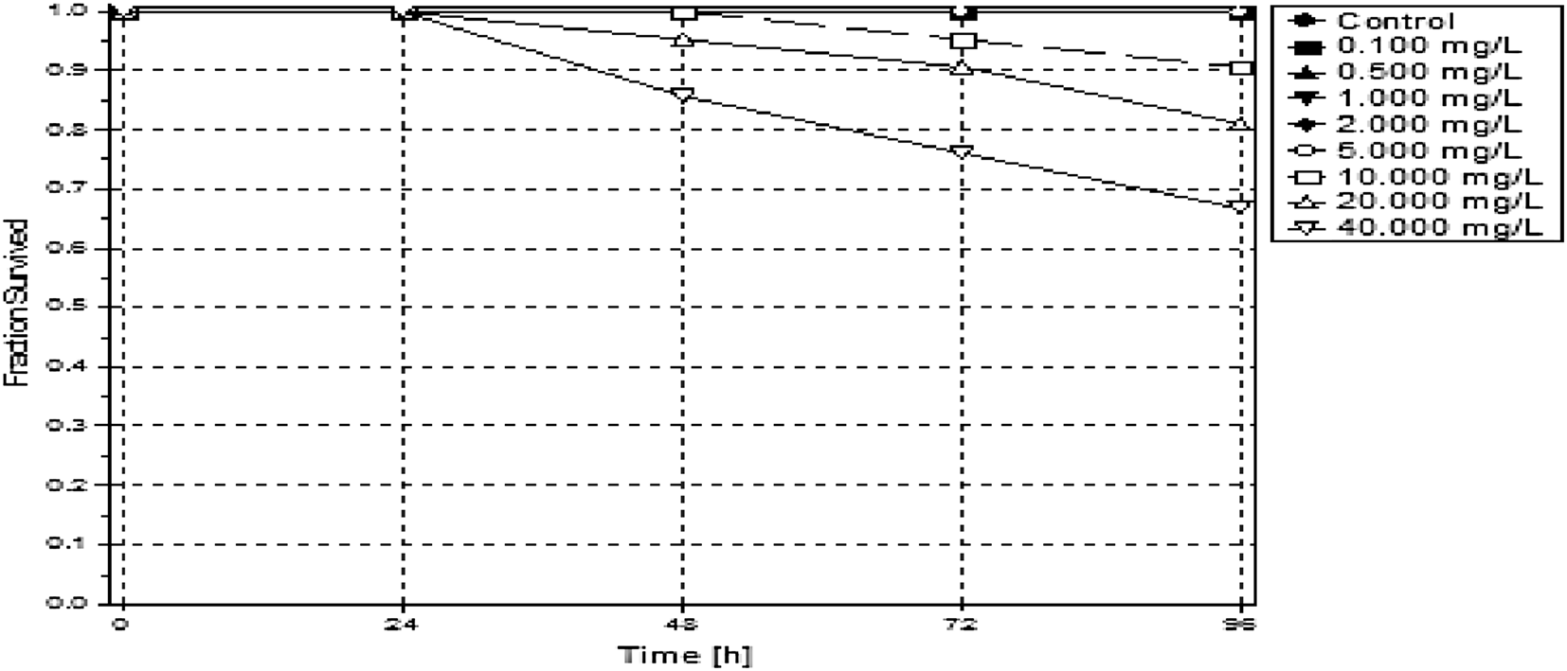
The survival of the introduced 14-day old *Danio rerio* as observed over a 96-h exposure to varying concentrations of conjugate.

There was a lower solubility observed during exposures of the LPV and the exposures sedimented out of solution from 20 ppm in the test beaker within 24 h. Methanol was not used as a dispersant in this study to maintain the integrity of the conjugate tested, and all other procedures were applied to both chemicals tested. The LPV had a tenfold higher LC50 (6.417 ppm) than the conjugate form (60.804 ppm) (Table 2). A dose-dependent toxicity was seen within the first 24 h of exposure. In comparison, the conjugate had lower toxicity observed after 48 h of exposure (Figs. 6, 7).

**Table 2:** Median lethal concentrations for 10, 20 and 50% (LC10, LC20, and LC50) effect and the corresponding 95% confidence intervals for test organisms exposed LPV conjugate.

	LC10	LC20	LC50
**LPV**			
Value [ppm]	n.d	0.050	6.417
Lower 95%-cl	n.d	n.d	2.038
Upper 95%-cl	n.d	0.302	49.157

	LC10	LC20	LC50
**Conjugate**			
Value [ppm]	13.357	22.473	60.804
Lower 95%-cl	5.729	14.009	35.821
Upper 95%-cl	20.305	39.592	307.323

**Figure 8:**
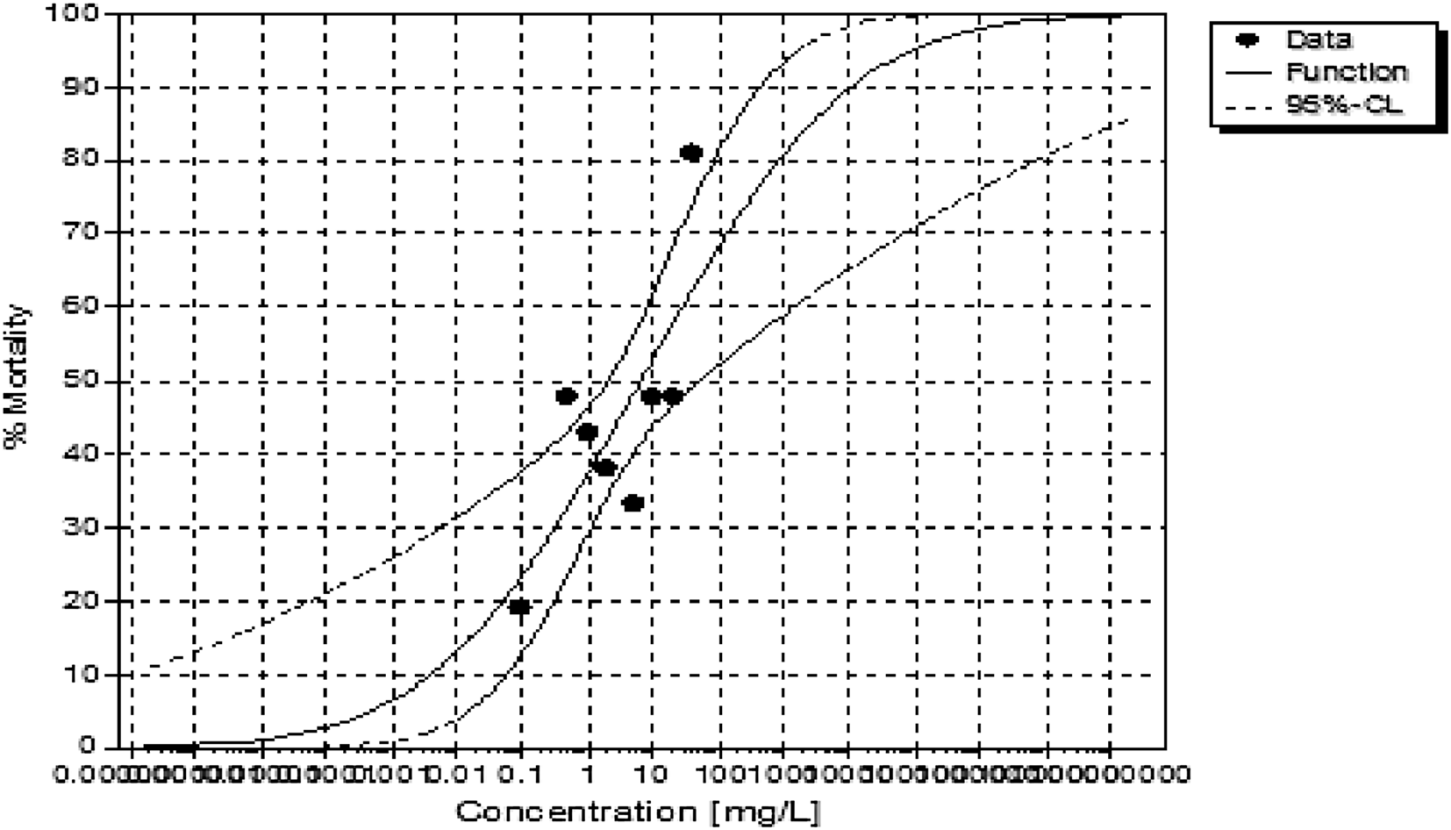
The concentration-effect curve used to calculate LCx values showing the influence of varying concentrations of LPV on *Danio rerio.*

**Figure 9:**
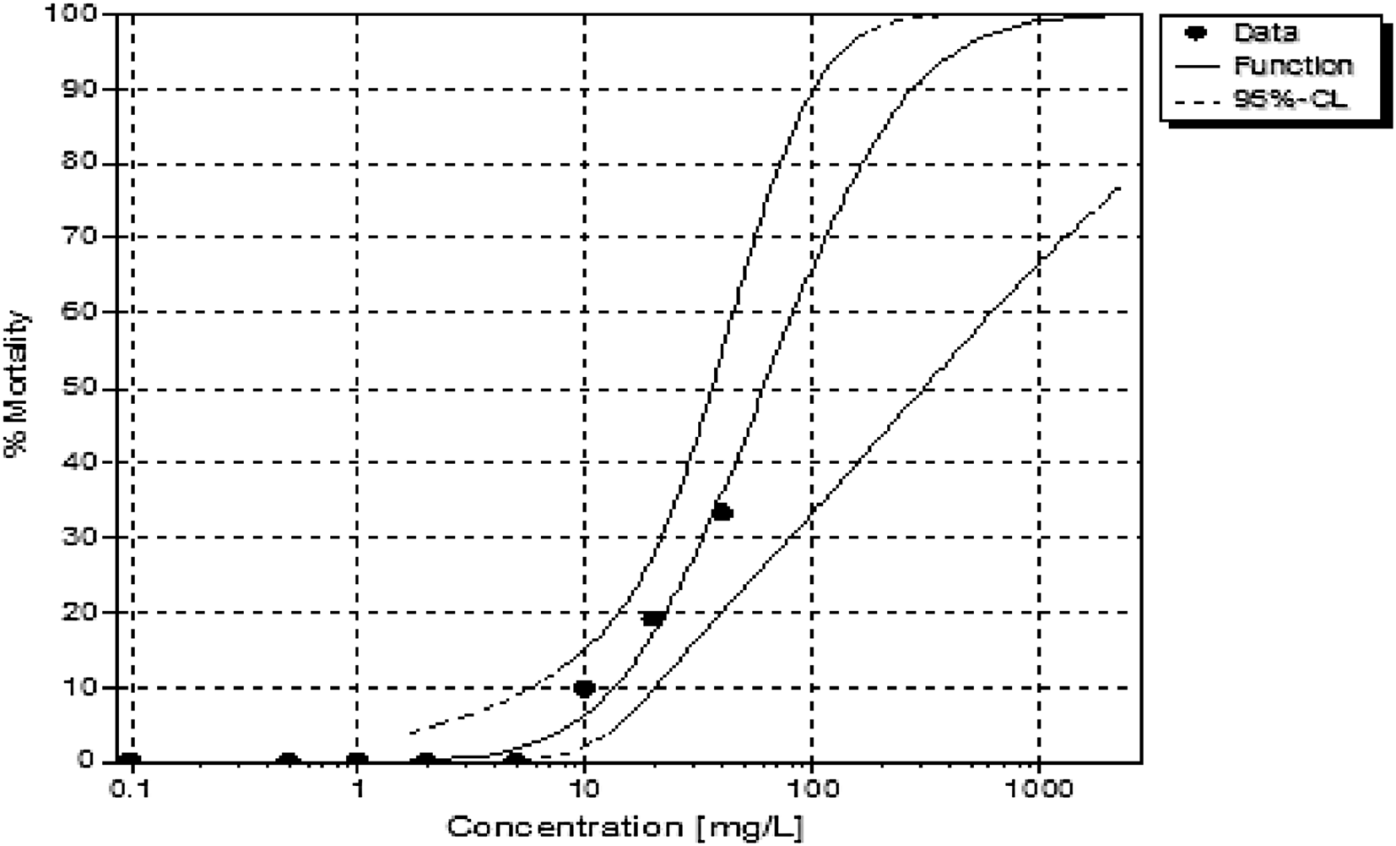
The concentration-effect curve used to calculate LCx values showing the influence of varying concentrations of the conjugate on *Danio rerio.*

The concentration-effect curves showed varied toxicity at 96 h in LPV, which can also be attributed to the low solubility, an effect that was not observed in the conjugate group (Figs. 8, 9). This is one of the first studies reporting acute toxicity of LPV and an LPV conjugate ARV using 14-day old *Danio rerio*.

## Conclusions

The study managed to synthesize the PEG–Suc–LPV conjugate successfully by using an esterification method, which indicates that this preparation method is a promising approach for establishing the solubility of LPV. The study further confirms that this increased solubility decreased the toxicity of LPV when used in a conjugate form after exposure to the *Danio rerio* Zebrafish assay.
